# CD8+ T cell-associated genes MS4A1 and TNFRSF17 are prognostic markers and inhibit the progression of colon cancer

**DOI:** 10.3389/fonc.2022.941208

**Published:** 2022-09-20

**Authors:** Ye Song, Zhipeng Zhang, Bo Zhang, Weihui Zhang

**Affiliations:** ^1^ Department of General Surgery, The First Affiliated Hospital of Harbin Medical University, Harbin, China; ^2^ Key Laboratory of Hepatosplenic Surgery, Ministry of Education, The First Affiliated Hospital of Harbin Medical University, Harbin, China

**Keywords:** MS4A1, TNFRSF17, colon cancer, CD8+ T cell, prognostic marker

## Abstract

**Background:**

Colon cancer (CC) is among the top three diseases with the highest morbidity and mortality rates worldwide. Its increasing incidence imposes a major global health burden. Immune checkpoint inhibitors, such as anti-PD-1 and anti-PD-L1, can be used for the treatment of CC; however, most patients with CC are resistant to immunotherapy. Therefore, identification of biomarkers that can predict immunotherapy sensitivity is necessary for selecting patients with CC who are eligible for immunotherapy.

**Methods:**

Differentially expressed genes associated with the high infiltration of CD8+ T cells were identified in CC and para-cancerous samples *via* bioinformatic analysis. Kaplan–Meier survival analysis revealed that MS4A1 and TNFRSF17 were associated with the overall survival of patients with CC. Cellular experiments were performed for verification, and the protein expression of target genes was determined *via* immunohistochemical staining of CC and the adjacent healthy tissues. The proliferation, migration and invasion abilities of CC cells with high expression of target genes were determined *via in vitro* experiments.

**Results:**

Differential gene expression, weighted gene co-expression and survival analyses revealed that patients with CC with high expression of MS4A1 and TNFRSF17 had longer overall survival. The expression of these two genes was lower in CC tissues than in healthy colon tissues and was remarkably associated with the infiltration of various immune cells, including CD8+ T cells, in the tumour microenvironment (TME) of CC. Patients with CC with high expression of MS4A1 and TNFRSF17 were more sensitive to immunotherapy. Quantitative reverse transcription-polymerase chain reaction, western blotting and immunohistochemical staining validated the differential expression of MS4A1 and TNFRSF17. In addition, Cell Counting Kit-8, wound healing and transwell assays revealed that the proliferation, migration and invasion abilities of CC cells were weakened after overexpression of MS4A1 and TNFRSF17.

**Conclusions:**

The core genes MS4A1 and TNFRSF17 can be used as markers to predict the sensitivity of patients with CC to immunotherapy and have potential applications in gene therapy to inhibit CC progression.

## Introduction

Colon cancer (CC) is one of the most prevalent malignant tumours worldwide and among the top three diseases with the highest mortality and morbidity rates (1). The pathogenesis of CC is complex, dynamic and unclear and can be attributed to the increase in income, changes in dietary structure and elevation in the number of patients with obesity and elderly people. In addition, genetic, mental and social factors play a role in its pathogenesis (2–4). At present, surgery, radiotherapy and chemotherapy are the standard treatment methods. However, due to the large trauma and easy recurrence of surgery, targeted therapy is becoming more and more popular. In addition, effective biomarkers and new intervention targets, which have clinical significance, are considered for the treatment of CC. Studies have shown that immune-related factors play a role in the occurrence and development of cancer. In addition to their function in clearance of lesions and inhibition of tumour occurrence, human immune cells play a role in immune cell formation in tumour tissues, therapeutic resistance, tumour invasion and other tumour-associated processes (5). For patients with stage I–III CC, T-cell infiltration has a greater prognostic value than TNM staging, and high CD8+ cell infiltration is a good prognostic marker for CC (6). During the anti-cancer immune response, CD8+ T cells play an important role in inducing tumour cell death by recognising tumour antigens. Immune checkpoint inhibitor (ICI)-based immunotherapy relies on the ability of CD8+ T cells to induce anti-tumour immune effects in CC, with tumour immune escape being manifested after the inhibition of CD8+ T cell activation (7, 8). The membrane-spanning 4-domains subfamily A 1 (MS4A1) and tumour necrosis factor receptor superfamily member 17 (TNFRSF17) genes are associated with immune infiltration and involved in the occurrence and development of several diseases, including CC(9-10).

MS4A1 (also called CD20) belongs to the membrane-spanning 4A gene family. It is a surface molecule found on B lymphocytes, which participates in the development and differentiation of B cells into plasma cells. CD20 is expressed throughout different B-cell developmental stages but is downregulated after B cells are differentiated into plasma cells. Therefore, MS4A1 serves as a marker for germinal centre-derived, naive and memory B cells (9). Although the role of MS4A1 in cancer remains unclear, bioinformatic studies have revealed the prognostic role of MS4A1 in various cancers and its correlation with immune cell infiltration. For example, MS4A1 can serve as an independent biomarker for predicting the prognosis of breast cancer, and its elevated expression is associated with a good prognosis (10). In gastric cancer, MS4A1 expression is upregulated, leading to a poor prognosis (11). Additionally, MS4A1 has been used to construct a prognostic model of lung cancer (12, 13). There have also been some studies on MS4A1in CC, Han et al. reported five potential gene biomarkers for predicting CC, including MS4A1, which was found to be downregulated (14). Most patients with CC are resistant to ICI immunotherapy; however, the underlying mechanisms remain unclear. Moreover, the decreased expression of MS4A1 in CC can be related to this resistance. Mudd Jr reported that MS4A1 expression was positively correlated with the survival of patients with CC. Additionally, CD20 expression was higher in anti-PD-1 antibody-bound T cells than in unbound T cells, suggesting that the CD8+CD20+ cytotoxic T lymphocyte (CTL) subset was the target of PD-L1-dependent immunosuppression in the human CC microenvironment. The loss of the CD8+CD20+ CTL subset in TME facilitates immune evasion of CC cells and their resistance to anti-PD-1 immunotherapy (15).

TNFRSF17(Tumour Necrosis Factor receptor superfamily member 17), also known as CD269 and B cell maturation antigen (BCMA), was first discovered in the early 1990s. It is a ubiquitously expressed plasmalemma antigen and a major target for T-cell therapy in multiple myeloma (16). The members of the TNFRSF superfamily participate in the immune response of the host, regulating cell proliferation, survival, differentiation and apoptosis. Stimulated by its ligands, TNFRSF17 activates mitogen-activated protein kinases and stimulates anti-apoptotic proteins, including Bcl-2 and Bcl-XL23, which generate signals that promote cell survival and proliferation (17). Although a study on breast cancer reported that NFRSF17 can act as a co-receptor of ALDH1A1, KLF4 and NANOG, mediating their tumour-promoting effects in breast cancer (18). However, the biological role of TNFRSF17 in other cancers remains unclear include CC. Chae et al. reported that TNFRSF17, found in resting and activated CD19+ cells, is a candidate gene responsible for the pathogenesis of inflammatory bowel disease. Additionally, the single nucleotide polymorphisms of TNFRSF17 can be associated with the tumour stage of CC (19). TNFRSF17 may also play an essential role in the pathogenesis of CC by influencing the immune response.

Human CC is among the rare human cancers that are resistant to ICI immunotherapy (20, 21). At present, non-responsiveness to ICI immunotherapy is a major challenge in human CC therapy (22–24). The expression of PD-L1 in TME correlates with the presence of CTLs, suggesting that defective CTL function in the CC microenvironment can be attributed to the non-responsiveness of CC to ICI immunotherapy. In this study, the genomic data of CC and healthy colon tissues were analysed *via* bioinformatic analysis, and two differential target genes closely related to immunity, MS4A1 and TNFRSF17, were identified. Although previous studies have reported that these genes are differentially expressed in CC and healthy colon tissues, their potential to predict the prognosis of CC remains unexplored. To the best of our knowledge, this study is the first to use bioinformatic analysis to screen these two genes as core genes associated with the high infiltration of CD8+ T cells in CC. Additionally, *in vitro* experiments were conducted to examine the effects of MS4A1 and TNFRSF17 on cancer proliferation, migration, invasion and other phenotypes. This study aimed to identify promising biomarkers for the prognosis of CC and the evaluation of immune checkpoint blockade efficacy, thereby identifying potential therapeutic targets for CC.

## Materials and methods

### Ethics statement

The study was approved by the Ethics Committee of The First Hospital Affiliated of Harbin University of Medicine (Heilongjiang, China) and performed in accordance with the principles of the Declaration of Helsinki.

### Public data source

**Figure 1:**
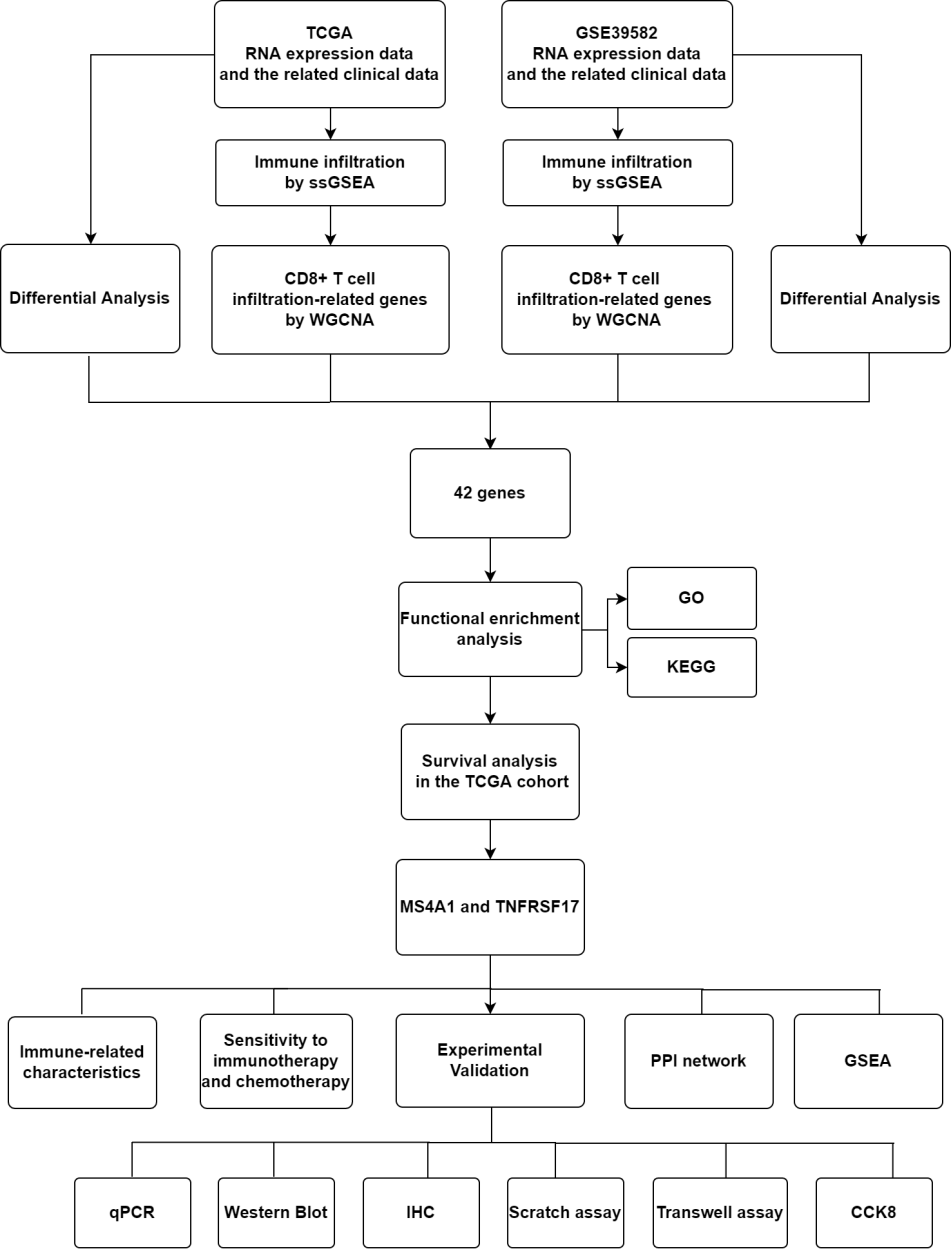
Flow Diagram of Experiment.

The flow chart of this experiment is shown in **Figure 1**. The RNA sequencing data (FPKM format) and clinical data of 455 CC samples and 41 healthy colon samples were retrieved from The Cancer Genome Atlas (TCGA) database (http://portal.gdc.cancer.gov/projects). The mRNA microarray data of 566 CC samples and 19 healthy colon samples were extracted from the GSE39582 dataset in the Gene Expression Omnibus (GEO) database (https://www.ncbi.nlm.nih.gov/geo/).

### Differential analysis

Differential mRNA expression of CC and healthy colon tissues in TCGA cohort was analysed using the ‘Limma’ package in the R software (version 4.0.2). Significantly differentially expressed genes were identified based on the fold change (FC) of ≥2 and corrected P-values (FDR) of ≤0.05. Differentially expressed genes in the GSE39582 dataset were analysed similarly.

### Weighted gene co-expression analysis

Co-expression networks were constructed using the ‘WGCNA’ package in R. CC samples in TCGA and GEO datasets were clustered to determine the presence of remarkable outliers. Subsequently, a co-expression network was developed using the automatic network construction function, and the soft threshold was computed using the pick Soft Threshold function. The co-expression similarity was derived according to the soft threshold, and the adjacency was calculated thereafter. Subsequently, the modules were examined using hierarchical clustering and dynamic tree-cut functions. Finally, gene significance and module membership were determined to correlate modules with the proportion of CD8+ T cells. Module genes with the strongest correlation were used for further analysis.

### Prognostic analysis

Overall survival (OS) and progression-free survival (PFS) were used to characterise the prognosis of patients with CC. Patients were divided into high- and low- expression groups based on the median gene expression. Kaplan–Meier survival analysis, log-rank test and the landmark were performed to examine differences in the survival of patients with CC between the two groups. A P-value of <0.05 was considered significant.

### Functional enrichment analysis

Gene ontology (GO) enrichment analysis is used for annotating functional genes, thereby analysing the molecular functions (MFs), associated biological pathways (BPs) and cellular components (CCs) of the target gene. Kyoto Encyclopedia of Genes and Genomes (KEGG) enrichment analysis is used for identifying the molecular functions and associated signalling pathways of the target gene. In this study, the ‘ClusterProfiler’ package in R was used to perform GO and KEGG enrichment analyses to better comprehend the biological function of target genes (P-values of <0.05 indicated significant enrichment). Gene set enrichment analysis (GSEA) based on KEGG analysis was performed using the GSEA software (version 4.1.0), with P-values of <0.05 and FDR of <0.25 indicating significant enrichment.

### Tumour stemness score

Malta et al. constructed the OCLR algorithm to calculate the RNA stemness scores of tumour samples in TCGA database based on the mRNA expression profile (mRNA expression-based stemness index [mRNAsi]) (25). Data regarding mRNAsi in TCGA-CC dataset were extracted from their study.

### Immune infiltration

The ESTIMATE algorithm is used for calculating not only immune but also stromal scores in TME based on mRNA expression. The immune and stromal scores reflect the total content of infiltrating immune cells and stroma in TME, respectively (26). In this study, the ‘ESTIMATE’ package in R was used to evaluate the immune and stromal scores of each sample in TCGA dataset. The ssGSEA algorithm, which can be used to calculate the infiltration scores of 24 types of immune cells and the immune status scores in TME based on mRNA expression, was used to evaluate the abundance of 24 types of immune cells and the immune status scores of CC samples in TCGA dataset. TISIDB (http://cis.hku.hk/TISIDB), which is an online database integrating various data resources of tumour immunology (27), was used to examine the relationship between target genes and various immune subtypes.

### Sensitivity to drug therapy

The TIDE algorithm is used to examine the sensitivity of patients to immunotherapy based on the mRNA expression of tumour samples (28). In this study, the algorithm was used to examine the sensitivity of patients with CC to immunotherapy in TCGA dataset. The higher the TIDE score, the worse the efficacy of immunotherapy and the shorter the survival time after receiving immunotherapy. The relationship between the expression of MS4A1 and TNFRSF17 and sensitivity to chemotherapeutic drugs was determined using the Gene Set Cancer Analysis (GSCA) database.

### GeneMANIA database

The GeneMANIA database (http://genemania.org/) was used to construct a protein–protein interaction (PPI) network, and the functions of proteins in the network were annotated.

### Immunohistochemical analysis

Immunohistochemical (IHC) staining was performed to verify the differential expression of target immune-related genes in cancer and adjacent tissues. Clinical samples were obtained from archived tissues of 5 patients with confirmed CC, who received surgical treatment in the First Affiliated Hospital of Harbin Medical University, and five adjacent healthy colon tissue samples. Malignant and paired adjacent tissues were embedded in paraffin, preparing two sets (5 versus 5) of 3-mm paraffin sections. Initially, these sections were deparaffinised and rehydrated before antigen retrieval. After blocking (Albumin Bovine, BioFroxx 4240, China) both CC and peri-lesional tissues were incubated with MS4A1 (Kit-0001, MXB, China) and TNFRSF17 (A01014-1, 1:50, BOSTER, China) primary antibodies overnight at 4**°**C. The following day, the sections were incubated with horseradish peroxidase-conjugated secondary antibody (IgG; PV-9000, ZSBIO, China) for 20 min at room temperature and washed thrice with phosphate-buffered solution. Each section was subsequently stained with 3,3′-diaminobenzidine and counterstained with haematoxylin. Membrane staining for MS4A1 and TNFRSF17 were considered as positive results at IHC. For each case, three 400-fold high-power fields were randomly observed for each index, and Mean Optical Density (MOD) value was calculated by Image Pro Plus (IPP) software.

### Cell culture and cell culture reagents

Human FHC cells (Fuheng Bio, China) were used as control cells, whereas SW480 and HCT116 cells (iCell Bioscience Inc, China) were used as experimental cells. FHC, SW480 and HCT116 cell lines were cultivated at 37°C in humidified conditions with 5% CO2 in Dulbecco’s Modified Eagle Medium (DMEM) (Gibco, USA) supplemented with 10% foetal bovine serum (FBS) (Gibco, USA), 100-U/mL penicillin and 100-μg/mL streptomycin (Gibco, USA).

### Transfection and grouping

After the cells reached 90% confluence, they were transfected with pCMV3-MS4A1 (Sino Biological, China), pCMV3-TNFRSF17 (Sino Biological, China) or pCMV3-untagged (Sino Biological, China) using Lipofectamine 2000 (Invitrogen, Thermo Fisher Scientific, USA). The two overexpression plasmids and empty plasmid were extracted using the endotoxin removal kit. After transfection, the cells were divided into the following six groups: 1) HCT116 + pCMV3-untagged, 2) HCT116 + pCMV3-MS4A1, 3) HCT116 + pCMV3-TNFRSF17, 4) SW480 + pCMV3-untagged, 5) SW480 + pCMV3-MS4A1 and 6) SW480 + pCMV3-TNFRSF17.

### RNA extraction and quantitative reverse transcription polymerase chain reaction

The three groups of cells (FHC, SW480 and HCT116) were not treated before transfection. Quantitative reverse transcription polymerase chain reaction (qRT-PCR) was performed to examine the RNA expression of MS4A1 in the HCT116 + pCMV3-untagged, HCT116 + pCMV3-MS4A1, SW480 + pCMV3-untagged, and SW480 + pCMV3-MS4A1 groups and the RNA expression of TNFRSF17 in the HCT116 + pCMV3-untagged, HCT116 + pCMV3-TNFRSF17, SW480 + pCMV3-untagged and SW480 + pCMV3-TNFRSF17 groups. GAPDH was used as an internal reference. Total RNA was extracted using the TRIzol reagent (Invitrogen, Thermo Fisher Scientific, USA) according to the manufacturer’s instructions. Briefly, 1 mL of TRIzol was added to a cell pellet (containing approximately 1x10^7^ cells), mixed and incubated at room temperature for 5 min. Subsequently, 0.2 mL of chloroform was added to the solution, vortexed for 15 sec and incubated for 3 min. To extract the supernatant, the cell lysate was subjected to centrifugation at 12,000 rpm and 4˚C for 10 min. The isolated supernatant was mixed with 0.5 mL of cold isopropanol and incubated on ice for 20–30 min. For RNA isolation, the solution was subjected to centrifugation at 12,000 rpm and 4˚C for 10 min. Subsequently, the supernatant was discarded, and the pellet was washed thrice with 1 ml of 75% ethanol. The RNA/ethanol mixture was subjected to centrifugation at 7,500 rpm for 5 min, and the supernatant was discarded. RNA was air−dried and dissolved in double-distilled water (ddH_2_O). The total volume of the qRT-PCR reaction mixture was 25 μL, and the mixture had the following components: 2 μL of RNA (template), 12.5 μL of SYBR^®^ PrimeScript Master Mix (2x, TOYOBO, China), 0.5 μL of forward primer (20 μM), 0.5 μL of reverse primer (20 μM) and 11.5 μL of ddH_2_O. GAPDH was used as an internal control. The qRT−PCR conditions were set as follows: 45˚C, 15 min; 95˚C, 5 min; 95˚C, 20 sec; 60˚C, 20 sec; 72˚C, 30 sec; 40 cycles. The following primers were used: human MS4A1 forward, TGATGCTGATCTTTGCCTTCTTCC; human MS4A1 reverse, TCGTCTCTGTTTCTTCTTCTTCCTC; human TNFRSF17 forward, CCATTCTTGTCACCACGAAAACG; human TNFRSF17 reverse, CTCTATCTCCGTAGCACTCAAAGC; human GAPDH forward, ACAACAGCCTCAAGATCATCAGC; human GAPDH reverse, GCCATCACGCCACAGTTTCC. The RNA expression levels were normalised to those of U6, and data were evaluated using the 2-ΔΔCq method.

### Western blotting

Western blotting (WB) was performed to examine the protein expression of MS4A1 in the HCT116 + pCMV3-untagged, HCT116 + pCMV3-MS4A1, SW480 + pCMV3-untagged and SW480 + pCMV3-MS4A1 groups (GAPDH was used as an internal reference). After 48 h of transfection, cells in the four groups were lysed. Subsequently, WB was performed to examine the protein expression of TNFRSF17 in the HCT116 + pCMV3-untagged, HCT116 + pCMV3-TNFRSF17, SW480 + pCMV3-untagged and SW480 + pCMV3-TNFRSF17 groups (GAPDH was used as an internal reference). Total protein was extracted and evaluated using WB following the standard procedure. Briefly, proteins were separated on 10% sodium dodecyl sulfate–polyacrylamide gels and transferred onto a nitrocellulose (NC) membrane. The NC membrane was incubated with a monoclonal primary antibody and blocked with tris-buffered saline with Tween (TBST) with 5% skimmed milk for 2 h and incubated overnight at 4˚C. The antibodies used were anti-TNFRSF17 (1:1000, Immunoway biotechnology, USA), anti-MS4A1 (1:1000, Immunoway biotechnology, USA) and anti-GAPDH (1:1000, Abcam, UK). Subsequently, the membrane was incubated with a secondary antibody at room temperature for 1.5 h and immersed in an enhanced chemiluminescence reagent for 1–3 min. Particular protein bands were visualised in the dark. Protein expression was evaluated *via* grey−value analysis using the ImagePro (version 5.0) software, and the relative expression level was evaluated as the ratio of target-to-reference protein expression.

### Cell proliferation assay using Cell Counting Kit−8

Cell proliferation was analysed using Cell Counting Kit−8 (Solarbio, China) according to the manufacturer’s instructions. Briefly, cells transfected with pCMV3-MS4A1, pCMV3-TNFRSF17 or the empty plasmid were seeded in 96−well culture plates (1,000 cells/well). The culture plates were incubated in a 5% CO_2_ incubator at 37°C for 0 h, 24 h, 48 h and 72 h. Subsequently, 10 uL of CCK−8 was added to cells, and absorbance was measured at 450 nm using a microplate reader. The experiment was performed in triplicate.

### Wound healing assay

All wells in a 24-well plate were coated with 0.1% gelatin solution (gelatin in deionised water) and incubated at 37°C for 5 h to prepare samples for wound healing assay. The coating solution was removed, and cells in the 6 groups were seeded in the plate at a density of 5000 cells/cm^2^ and incubated at 37°C (5% CO_2_) until a confluent cell monolayer was formed in every well. The medium was replaced with DMEM on the following day, and the cells were incubated with 30-μM mitomycin C for 72 h. Thereafter, a scratch was made in the cell monolayer with the tip of a pipette, and a cell-free region was obtained by removing detached cells from the cell monolayer.

### Transwell assay

Approximately 60 µL of diluted matrigel (1:8, BD, USA) was added to the upper transwell chamber, and the plate was incubated for 4–5 h. After the gel had solidified, the residual liquid was aspirated, and 100 µL of serum-free DMEM was added. The chamber was incubated and hydrated for 20 min. To prepare a cell suspension, cells were digested and resuspended in serum-free DMEM and quantified using a haemocytometer. The upper chamber was inoculated with 1×10^4^ cells, whereas the lower chamber was inoculated with DMEM containing 10% FBS, and the plate was incubated in a 5% CO_2_ incubator at 37°C. After 24 h, the medium in the well plate was aspirated, and the cells were fixed with 4% paraformaldehyde for 15 min. After 20 min of crystal violet staining, the residual crystal violet solution was washed several times with PBS, and a microscope was used to capture images of the cells.

### Statistical analyses

The independent samples t-test, Kruskal–Wallis test, one-way analysis of variance, Mann–Whitney test and Wilcoxon signed-rank test were used to examine differences in continuous variables between groups. The chi-square or Fisher’s test was used to analyse categorical variables. Survival analysis was performed using the Kaplan–Meier method, log-rank test and landmark. Correlation analysis was performed using Pearson’s (normal distribution) and Spearman’s (abnormal distribution) correlation tests. To ensure accuracy, all experiments were performed in triplicate. A P-value of <0.05 denoted significant differences (*, <0.05; **, <0.01; ***, <0.001; ns, >0.05).

## Results

### Differential analysis

Differences in mRNA expression between CC and healthy colon samples in TCGA and GEO databases were analysed. A total of 3,841 differentially expressed mRNAs were identified from CC samples in TCGA dataset; of which, 1,208 and 2,273 were upregulated and downregulated, respectively, in CC. A total of 1,460 differentially expressed mRNAs were identified from CC samples in the GSE39582 dataset; of which, 665 and 795 were upregulated and downregulated, respectively, in CC. Heat maps are shown in **Supplementary Figures 1A, B**, and volcano plots are shown in **Supplementary Figures 1C, D**.

### Weighted gene co-expression analysis

CD8+ T cells play a substantial role in anti-cancer immunity, and high infiltration of CD8+ T cells can often effectively kill tumour cells. Therefore, genes associated with the high infiltration of CD8+ T cells were identified. A total of 7 gene co-expression modules were identified based on CC samples in TCGA dataset **(** **Supplementary Figure 2A** **)**, and 14 gene co-expression modules were identified based on CC samples in the GEO dataset **(** **Supplementary Figure 2B** **)**. The brown modules of both TCGA and GEO datasets were significantly positively correlated with high infiltration of CD8+ T cells **(** **Supplementary Figures 2C, D** **)** (TCGA: correlation coefficient, 0.46, P < 0.05; GEO: correlation coefficient, 0.57, P < 0.05). The brown module of TCGA data and GEO data contained 2956 genes and 445 genes, respectively, which are significantly linked to the high infiltration of CD8+T cells. To screen out genes that were linked to both the occurrence as well as the progression of CC and the high infiltration of CD8+T cells. The 42 genes, which were intersected by the four data sets in the flow chart, both dysregulated in CC and associated with CD8+T cell infiltration in the TCGA and GEO cohorts **(** **Figure 1** and **Supplementary Figure 2E** **)**.

### Functional enrichment analysis

**Figure 2:**
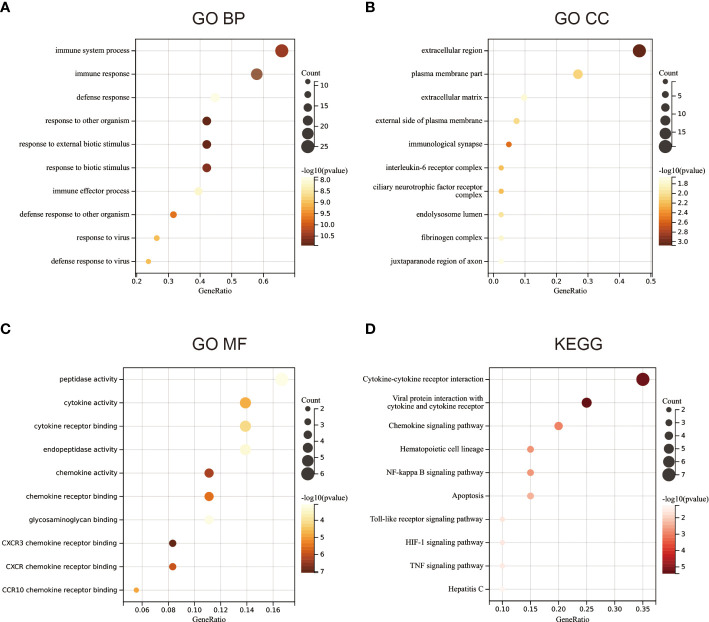
Functional enrichment analysis. **(A)** Gene ontology (GO) enrichment analysis of biological processes. **(B)** GO enrichment analysis of cellular components. **(C)** GO enrichment analysis of molecular functions. **(D)** KEGG functional enrichment analysis.

To examine the biological role of the 42 candidate genes, GO and KEGG enrichment analyses were performed. GO enrichment analysis revealed that the 42 candidate genes were mainly associated with BPs, including immune response and antiviral immunity (**Figure 2A**), especially the formation of the extracellular matrix (**Figure 2B**) and chemokine and cytokine activity and binding (**Figure 2C**). KEGG functional enrichment analysis revealed that the genes were mainly involved in the NF-KB signalling pathway, cytokine–cytokine receptor interaction, toll-like receptor signalling pathway, chemokine signalling pathway, tumour necrosis factor signalling pathway and other signalling pathways (**Figure 2D**).

### MS4A1 and TNFRSF17 are associated with the prognosis of CC and stemness scores

**Figure 3:**
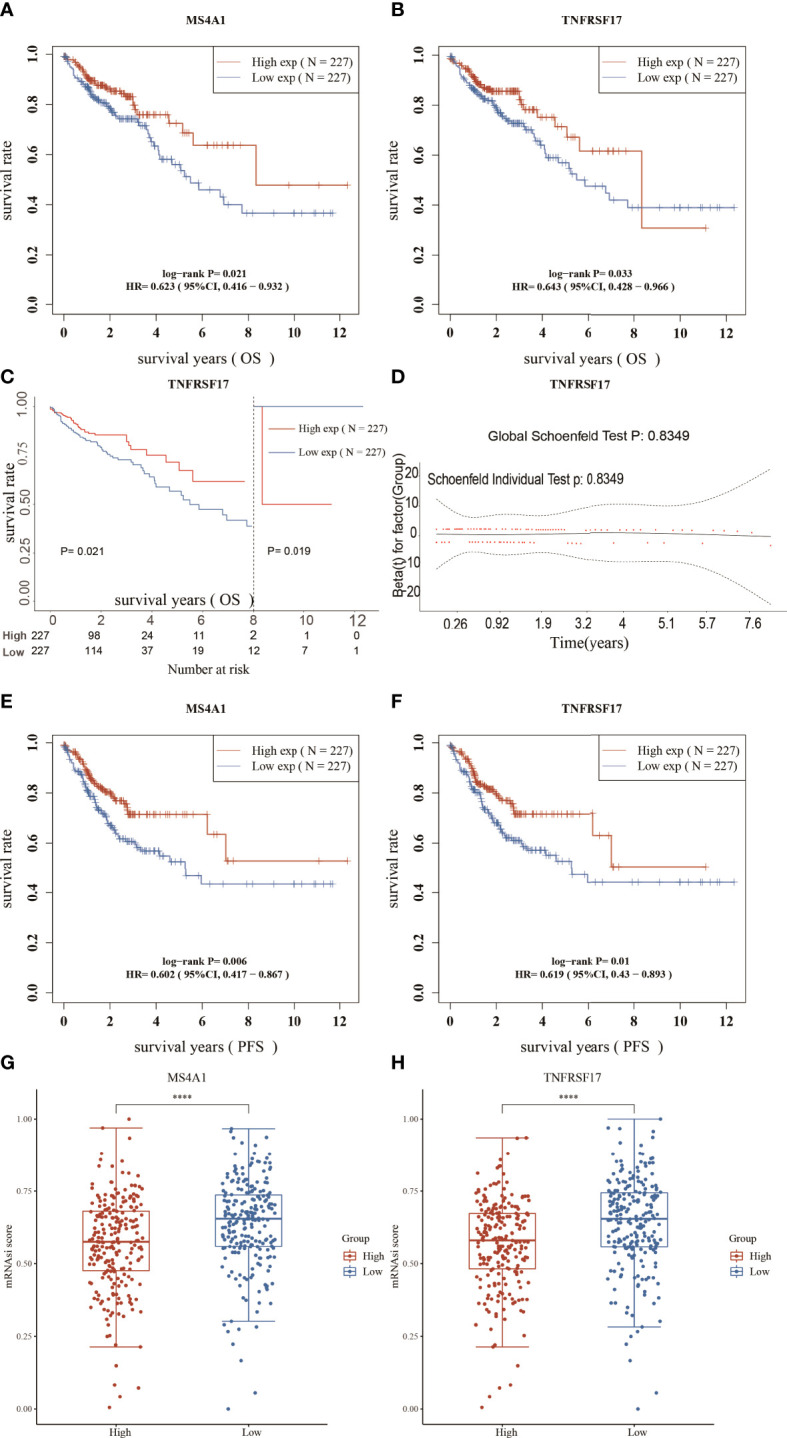
Survival analysis and stemness scores. **(A)** Overall survival (OS) analysis of the high- and low-MS4A1-expression groups. **(B)** OS analysis of the high- and low-TNFRSF17-expression groups. **(C)** Landmark analysis results **(D)** Proportional risk assumption results **(E)** Progression-free survival (PFS) analysis of the high- and low-MS4A1-expression groups. **(F)** PFS survival analysis of the high- and low-TNFRSF17-expression groups. **(G)** Stemness scores of the high- and low-MS4A1-expression groups. **(H)** Stemness scores of the high- and low-TNFRSF17-expression groups. ****,<0.0001.

Survival analysis of the 42 candidate genes was performed. Patient samples with insufficient survival data in TCGA dataset were removed, and 454 samples were eventually included in analysis. Survival analysis showed that among the 42 genes, only MS4A1 and TNFRSF17 were associated with OS in CC. The expression of both genes was low in CC **(** **Supplementary Figures 1A–D** **)**, and OS and PFS were shorter in patients with low expression of the two genes than in patients with high expression of the two genes **(** **Figures 3A–F** **)**, suggesting that these two genes can act as tumour suppressor genes, and their expression was suppressed in CC. The stemness score is an indicator of tumour malignancy, and the higher the stemness score, the more aggressive the tumour (25). Patients with CC with high MS4A1 and TNFRSF17 expression had lower stemness scores **(** **Figures 3G, H** **)**, which further indicates that MS4A1 and TNFRSF17 can act as tumour suppressor genes.

### Expression of MS4A1 and TNFRSF is lower in CC and related to clinical features

**Figure 4:**
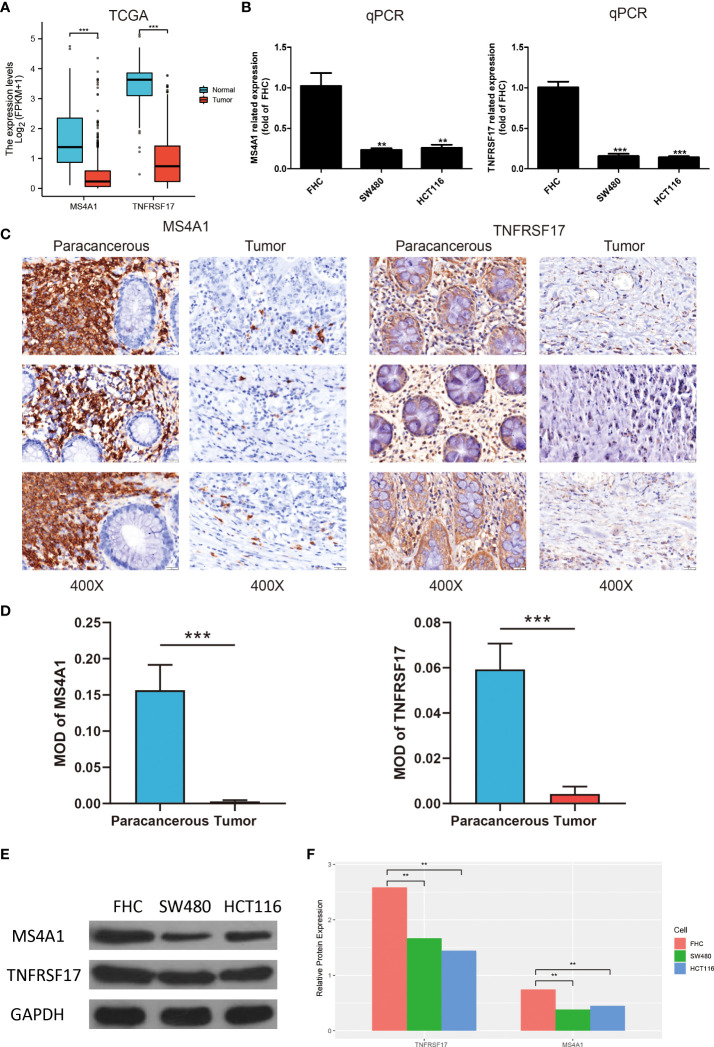
Expression of MS4A1 and TNFRSF is lower in colon cancer (CC) and related to clinical features. **(A)** Expression of MS4A1 and TNFRSF17 at the transcription level was lower in colon cancer (CC) tissues than in healthy colon tissues. **(B)** qRT-PCR showed that the mRNA expression of MS4A1 and TNFRSF17 was higher in FHC cells than in SW480 and HCT116 cells. **(C)** Matched pairs of CC tissues and adjacent non-neoplastic colon tissues were stained with MS4A1- and TNFRSF17-specific antibodies for immunohistochemical analysis. Representative images of tissues and tumour-infiltrating cells are shown (scale bar = 20 μm). **(D)** The MOD of MS4A1 and TNFRSF17 is obtained by analysing the photo optical density with IPP software. **(E, F)** Western blotting revealed that the protein expression of MS4A1 and TNFRSF17 was higher in FHC cells than in SW480 and HCT116 cells. **,<0.01; ***,<0.001.

The expression of MS4A1 and TNFRSF17 was lower at the transcriptional level in CC samples than in healthy colon tissues in TCGA dataset (**Figure 4A**). qRT-PCR analysis of different cell lines revealed consistent results (**Figure 4B**). Immunohistochemical analysis showed that MS4A1 and TNFRSF17 positive cells were leukocytes, and were mainly located in the cells within the lymphocyte structure. At higher magnification, the protein expression of MS4A1 and TNFRSF17 was lower in CC than in adjacent peri-lesional normal tissues (**Figure 4C** **)**. The MOD of MS4A1 and TNFRSF17 showed that the expression of MS4A1 and TNFRSF17 in the cell membrane of colon cancer was significantly lower than that of adjacent tissues (*P*<0.001, **Figure 4D**). Similarly, WB of the cell lines provided consistent results (**Figures 4E, F**). Furthermore, the relationship between the clinical information of patients with CC in TCGA cohort and the expression of MS4A1 and TNFRSF17 was analysed. The results showed that patients with CC with high MS4A1 expression had early T- and pathological-stage CC and a more frequent history of colon polyps (**Table 1**). Patients with CC having a high expression of TNFRSF17 showed some differences in the M stage of CC and history of colon polyps. When TNFRSF17 expression was high, The proportion of distant metastasis was lower, but the proportion of history of colon polyps was higher (**Table 2**).

**Table 1:** Analysis of correlation between expression of MS4A1 and clinical.

Characteristic	Low expression of MS4A1	High expression of MS4A1	p
n	227	227	
T stage, n (%)			0.011
T1	2 (0.4%)	9 (2%)	
T2	29 (6.4%)	48 (10.6%)	
T3	165 (36.4%)	144 (31.8%)	
T4	31 (6.8%)	25 (5.5%)	
N stage, n (%)			0.471
N0	129 (28.4%)	138 (30.4%)	
N1	58 (12.8%)	47 (10.4%)	
N2	40 (8.8%)	42 (9.3%)	
M stage, n (%)			0.130
M0	155 (39%)	178 (44.8%)	
M1	37 (9.3%)	27 (6.8%)	
Pathologic stage, n (%)			0.012
Stage I	25 (5.6%)	50 (11.3%)	
Stage II	94 (21.2%)	82 (18.5%)	
Stage III	67 (15.1%)	61 (13.8%)	
Stage IV	37 (8.4%)	27 (6.1%)	
Primary therapy outcome, n (%)			0.283
PD	11 (4.7%)	14 (6%)	
SD	0 (0%)	4 (1.7%)	
PR	7 (3%)	5 (2.1%)	
CR	88 (37.4%)	106 (45.1%)	
Gender, n (%)			1.000
Female	107 (23.6%)	107 (23.6%)	
Male	120 (26.4%)	120 (26.4%)	
Weight, n (%)			0.540
<=90	96 (38.6%)	80 (32.1%)	
>90	36 (14.5%)	37 (14.9%)	
Height, n (%)			0.591
<170	62 (26.7%)	52 (22.4%)	
>=170	59 (25.4%)	59 (25.4%)	
BMI, n (%)			0.433
<25	44 (19%)	34 (14.7%)	
>=25	77 (33.2%)	77 (33.2%)	
Residual tumor, n (%)			0.729
R0	154 (43.3%)	174 (48.9%)	
R1	1 (0.3%)	3 (0.8%)	
R2	12 (3.4%)	12 (3.4%)	
CEA level, n (%)			0.952
<=5	88 (30.8%)	100 (35%)	
>5	47 (16.4%)	51 (17.8%)	
Perineural invasion, n (%)			1.000
NO	67 (37.4%)	66 (36.9%)	
YES	23 (12.8%)	23 (12.8%)	
Lymphatic invasion, n (%)			0.258
NO	128 (31.1%)	120 (29.2%)	
YES	74 (18%)	89 (21.7%)	
History of colon polyps, n (%)			0.031
NO	133 (34.5%)	117 (30.3%)	
YES	56 (14.5%)	80 (20.7%)	
Colon polyps present, n (%)			0.412
NO	80 (35.6%)	66 (29.3%)	
YES	38 (16.9%)	41 (18.2%)	
Neoplasm type, n (%)			1.000
Colon adenocarcinoma	227 (50%)	227 (50%)	
Rectum adenocarcinoma	0 (0%)	0 (0%)	
Age, meidan (IQR)	69 (58, 77)	68 (58, 77.5)	0.972

**Table 2:** Analysis of correlation between expression of TNFRSF17 and clinical.

Characteristic	Low expression of TNFRSF17	High expression of TNFRSF17	p
n	227	227	
T stage, n (%)			0.383
T1	3 (0.7%)	8 (1.8%)	
T2	40 (8.8%)	37 (8.2%)	
T3	153 (33.8%)	156 (34.4%)	
T4	31 (6.8%)	25 (5.5%)	
N stage, n (%)			0.348
N0	128 (28.2%)	139 (30.6%)	
N1	59 (13%)	46 (10.1%)	
N2	40 (8.8%)	42 (9.3%)	
M stage, n (%)			0.028
M0	155 (39%)	178 (44.8%)	
M1	40 (10.1%)	24 (6%)	
Pathologic stage, n (%)			0.161
Stage I	35 (7.9%)	40 (9%)	
Stage II	82 (18.5%)	94 (21.2%)	
Stage III	64 (14.4%)	64 (14.4%)	
Stage IV	40 (9%)	24 (5.4%)	
Primary therapy outcome, n (%)			0.426
PD	11 (4.7%)	14 (6%)	
SD	1 (0.4%)	3 (1.3%)	
PR	8 (3.4%)	4 (1.7%)	
CR	86 (36.6%)	108 (46%)	
Gender, n (%)			0.925
Female	106 (23.3%)	108 (23.8%)	
Male	121 (26.7%)	119 (26.2%)	
Weight, n (%)			0.677
<=90	96 (38.6%)	80 (32.1%)	
>90	37 (14.9%)	36 (14.5%)	
Height, n (%)			0.885
<170	61 (26.3%)	53 (22.8%)	
>=170	61 (26.3%)	57 (24.6%)	
BMI, n (%)			0.332
<25	45 (19.4%)	33 (14.2%)	
>=25	77 (33.2%)	77 (33.2%)	
Residual tumor, n (%)			0.776
R0	151 (42.4%)	177 (49.7%)	
R1	2 (0.6%)	2 (0.6%)	
R2	13 (3.7%)	11 (3.1%)	
CEA level, n (%)			0.100
<=5	81 (28.3%)	107 (37.4%)	
>5	53 (18.5%)	45 (15.7%)	
Perineural invasion, n (%)			0.405
NO	75 (41.9%)	58 (32.4%)	
YES	22 (12.3%)	24 (13.4%)	
Lymphatic invasion, n (%)			0.546
NO	119 (29%)	129 (31.4%)	
YES	84 (20.4%)	79 (19.2%)	
History of colon polyps, n (%)			0.026
NO	134 (34.7%)	116 (30.1%)	
YES	56 (14.5%)	80 (20.7%)	
Colon polyps present, n (%)			1.000
NO	77 (34.2%)	69 (30.7%)	
YES	42 (18.7%)	37 (16.4%)	
Neoplasm type, n (%)			1.000
Colon adenocarcinoma	227 (50%)	227 (50%)	
Rectum adenocarcinoma	0 (0%)	0 (0%)	
Age, meidan (IQR)	69 (58, 77.5)	68 (59, 77)	0.577

### MS4A1 and TNFRSF17 are associated with immune infiltration

**Figure 5:**
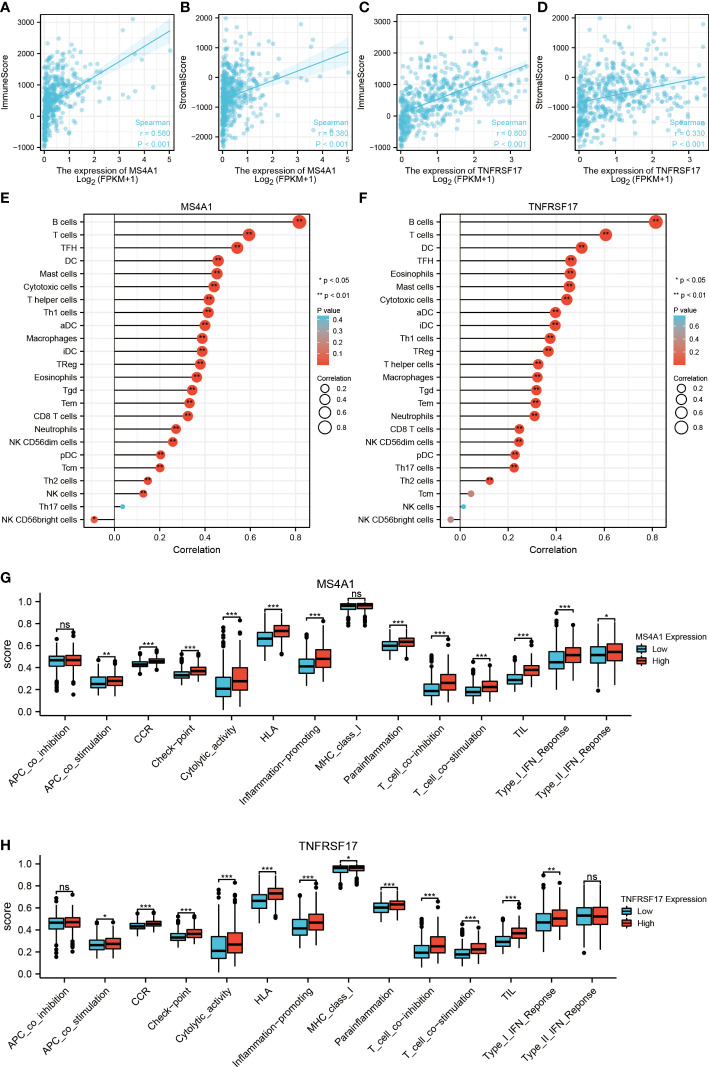
Relationship between the target genes and immune infiltration. **(A)** Correlation between MS4A1 expression and immune scores. **(B)** Correlation between MS4A1 expression and stromal scores. **(C)** Correlation between TNFRSF17 expression and immune scores. **(D)** Correlation between TNFRSF17 expression and stromal scores. **(E)** Relationship between MS4A1 expression and immune cell infiltration in TME. **(F)** Correlation between TNFRSF17 expression and immune cell infiltration in TME. **(G)** Comparison of the immune status between the high- and low-MS4A1-expression groups. **(H)** Comparison of the immune status between the high- and low-TNFRSF17-expression groups. *,<0.05; **,<0.01; ***,<0.001; ns,>0.05.

Previous studies have reported that MS4A1 and TNFRSF17 are closely related to the immune response of CC. Therefore, the relationship between the TME of CC and the expression of MS4A1 and TNFRSF17 was systematically analysed. The expression of MS4A1 (*P* < 0.05, R > 0.3) and TNFRSF17 (*P* < 0.05, R > 0.3) was positively correlated with immune scores and immune cell infiltration in the TME of CC **(** **Figures 5A–D** **)**. In addition, the expression of MS4A1 and TNFRSF17 was positively correlated with immune cell infiltration and matrix components in the TME of CC. The immune scores can only reflect the total amount of immune cells infiltrated in TME but not the immune status of TME. Therefore, the ssGSEA algorithm was used to evaluate the correlation between the expression of MS4A1 and TNFRSF17 and the infiltration of 24 types of immune cells in the TME of CC. The results revealed that MS4A1 expression was positively correlated with the infiltration of B cells, central memory T cells, T cells, T follicular helper cells, dendritic cells, mast cells, cytotoxic cells, T helper cells, Th1 cells, activated dendritic cells, macrophages, immature dendritic cells, regulatory T cells, eosinophils, gammadelta T cells, effector memory T cells, cytotoxic T cells, neutrophils, CD56^dim^ natural killer cells, plasmacytoid dendritic cells, Th2 cells and natural killer cells but negatively correlated with the infiltration of CD56^bright^ natural killer cells (*P* < 0.05, **Figure 5E**). TNFRSF17 expression was positively correlated with the infiltration of B cells, T cells, T follicular helper cells, dendritic cells, mast cells, cytotoxic cells, T helper cells, Th1 cells, activated dendritic cells, central memory T cells, macrophages, immature dendritic cells, regulatory T cells, eosinophils, gammadelta T cells, effector memory T cells, cytotoxic T cells, neutrophils, natural killer cells CD 56^dim^ cells, plasmacytoid dendritic cells and Th2 cells (*P* < 0.05, **Figure 5F**). Furthermore, the immune status was compared between the two expression groups of MS4A1 and TNFRSF17. Immune checkpoint scores, cytolytic toxicity and various immune response scores were higher in TME in the high-MS4A1-expression group than in the low-MS4A1-expression group (*P* < 0.05, **Figure 5G**). Similar results were obtained for TNFRSF17 (**Figure 5H**). Therefore, the infiltration of various types of immune cells in TME increases owing to the high expression of MS4A1 and TNFRSF17 in CC, and the immune response is more active.

### MS4A1 and TNFRSF17 are associated with CC drug sensitivity

**Figure 6:**
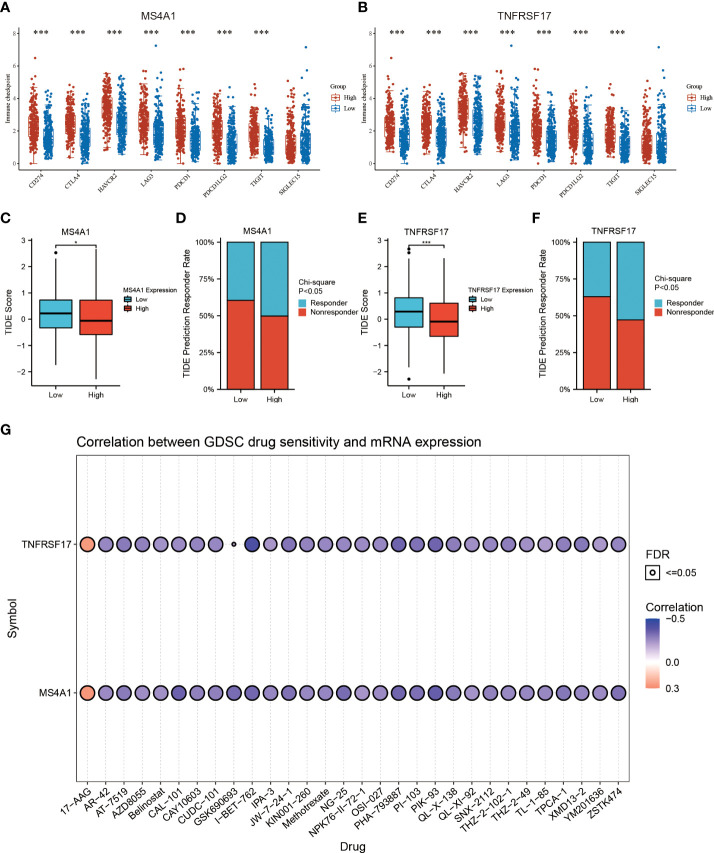
MS4A1 and TNFRSF17 are associated with CC drug sensitivity. **(A)** Differences in the expression of common immune checkpoints between the high- and low-MS4A1-expression groups. **(B)** Differences in the expression of common immune checkpoints between the high- and low-TNFRSF17-expression groups. **(C)** Differences in TIDE scores between the high- and low-MS4A1-expression groups. **(D)** Differences in response rates predicted based on TIDE scores between the high- and low-MS4A1-expression groups. **(E)** Differences in TIDE scores between the high- and low-TNFRSF17-expression groups. **(F)** Differences in response rates predicted based on TIDE scores between the high- and low-TNFRSF17-expression groups. **(G)** Correlation between drug sensitivity and gene expression. *, <0.05; ***, <0.001.

Recently, immunotherapy has made great progress in cancer treatment. Some ICIs, such as anti-PD-1 and anti-PD-L1, have been validated for use in the treatment of cancer. However, most patients with CC lack sensitivity to immunotherapy (16). Therefore, identification of biomarkers for predicting sensitivity to immunotherapy is crucial for the selection of patients with CC who are eligible for immunotherapy. In this study, differences in common immune checkpoints between the two expression groups of MS4A1 and TNFRSF17 were analysed. The results revealed that the expression of PD-L1 (CD274), CTLA4, HAVCR2, LAG3, PD1 (PDCD1), PDCD1LG2, TIGIT and other immune checkpoints was higher in the high-MS4A1-expression and high-TNFRSF17-expression groups than in the low-MS4A1-expression and low-TNFRSF17-expression groups (*P* < 0.05, **Figures 6A, B**). These results suggest that patients with CC with high expression of MS4A1 and TNFRSF17 can benefit from ICI immunotherapy. To further examine the association between the expression of MS4A1 and TNFRSF17 and sensitivity to immunotherapy, differences in TIDE scores between the two expression groups of MS4A1 and TNFRSF17 were analysed. The TIDE score is used to examine the response of patients to immunotherapy. The lower the score, the higher the sensitivity to immunotherapy. In the high-MS4A1-expression group, the TIDE scores of patients with CC were lower, whereas the proportion of patients who responded to immunotherapy was higher (*P* < 0.05, **Figures 6C, D**). Similar results were observed in the high-TNFRSF17-expression group (*P* < 0.05, **Figures 6E, F**). These results indicate that patients with CC with high MS4A1 and TNFRSF17 expression are more sensitive to immunotherapy. Furthermore, the relationship between the expression of MS4A1 and TNFRSF17 and chemotherapeutic drug sensitivity was examined. **Figure 6G** shows the drugs for which chemosensitivity was remarkably associated with the expression of MS4A1 and TNFRSF17. In addition to being insensitive to 17-AAG, patients with high expression of MS4A1 and TNFRSF17 showed high sensitivity to other chemotherapeutic drugs.

### PPI and functional enrichment analysis of MS4A1 and TNFRSF17

According to the established PPI network, the proteins interacting with MS4A1 and TNFRSF17 were TNFSF13, POU2F2, TNFSF13B, TRAF5, TRAF1, CR2, CD53, TRAF3, POU2AF1, GPR18, CD22, CD40, CD79B, CD27, TCL1A, JCHAIN, IRF8, OTULINL, CD72 and IRAG2. The main functions of these proteins are B-cell activation, lymphocyte proliferation, response to tumour necrosis factor, leukocyte proliferation, positive regulation of stress-activated MAPK cascade and lymphocyte differentiation (**Supplementary Figure 3A**). Additionally, GSEA showed that MS4A1 and TNFRSF17 were mainly related to the chemokine signalling pathway, cytokine receptor interaction, T cell receptor signalling pathway, natural killer cell-mediated cytotoxicity and B cell receptor signalling pathway (**Supplementary Figures 3B, C**).

### Construction and identification of overexpressing cell lines

**Figure 7:**
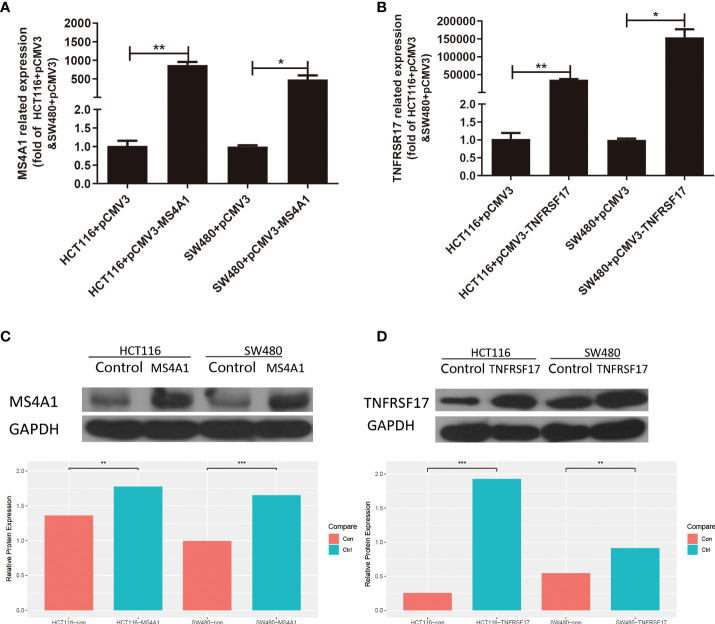
Expression changes after transfection of MS4A1 and TNFRSF17. **(A, B)** Compared with the empty negative control group, both MS4A1 and TNFRSF17 gene overexpression plasmids transfected into SW480 and HCT116 cells, the RNA levels detected by QPCR were overexpressed **(C, D)** Compared with the empty negative control group, both MS4A1 and TNFRSF17 gene overexpression plasmids were transfected into SW480 and HCT116 cells, and the protein levels detected by WB had overexpression effects. *,<0.05; **,<0.01; ***,<0.001.

After transfection, the overexpressed CC cell lines were identified, and the transfection efficiency of MS4A1 and TNFRSF17 overexpression plasmids in SW480 and HCT116 cells was analysed *via* qRT-PCR and WB, respectively. Compared with the negative control group, SW480 and HCT116 cells transfected with MS4A1 and TNFRSF17 overexpression plasmids had increased expression of both genes at the RNA level (*P* < 0.05, **Figures 7A, B**). Subsequently, protein expression was analysed *via* WB. After the MS4A1 and TNFRSF17 overexpression plasmids were transfected into SW480 and HCT116 cells, the expression of both genes was increased at the protein level as well (*P* < 0.01, **Figures 7C, D**). These results indicated that cell lines with MS4A1 and TNFRSF17 overexpression were successfully established, and subsequent functional experiments were performed using them.

### MS4A1 and TNFRSF17 inhibited CC progression *in vitro*

**Figure 8:**
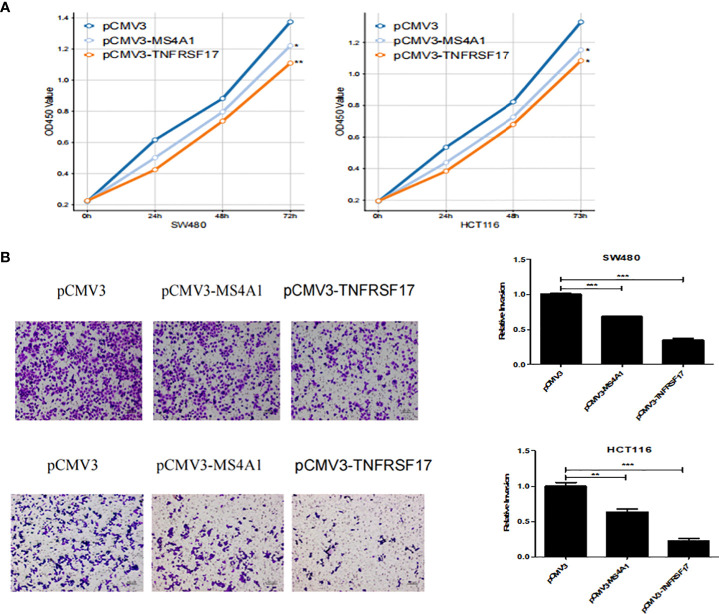
CCK8 and Transwell experiments. **(A)** The proliferation ability of SW480 cells and HCT116 cells transfected with pCMV3-MS4A1 and pCMV3-TNFRSF17 was significantly reduced. **(B)** The invasive ability of SW480 cells and HCT116 cellstransfected with pCMV3-MS4A1 and pCMV3-TNFRSF17 was significantly reduced. *,<0.05; **,<0.01; ***,<0.001.

**Figure 9:**
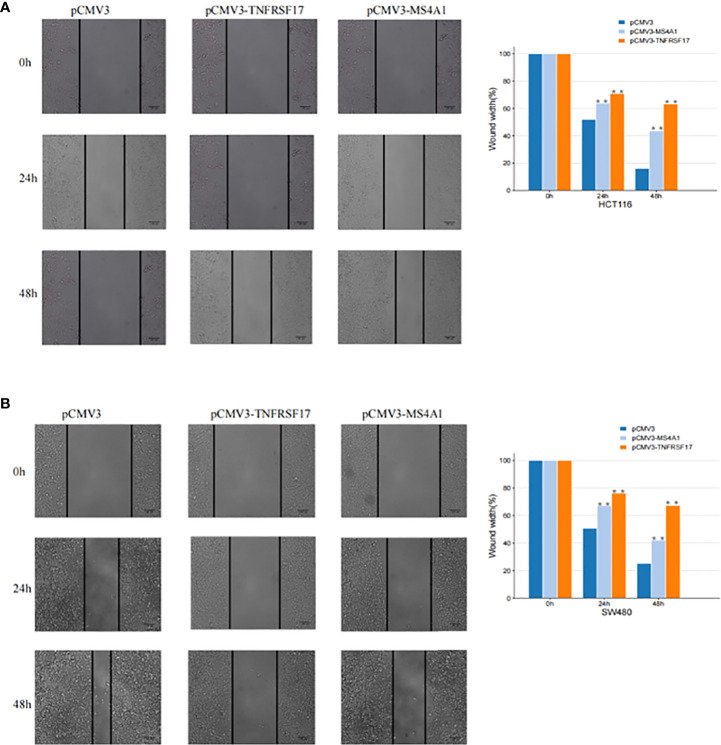
Cell scratch experiment. **(A)** The migration ability of SW480 cells transfected with pCMV3-MS4A1 and pCMV3-TNFRSF17 was significantly weakened. **(B)** The migration ability of HCT116 cells was significantly reduced after pCMV3-MS4A1 and pCMV3-TNFRSF17 were transfected. *,<0.05.

To examine the effects of MS4A1 and TNFRSF17 on the proliferation, invasion and migration abilities of CC cells, CCK8, transwell invasion and wound healing assays were performed with cell lines overexpressing MS4A1 and TNFRSF17 and negative control groups. CCK8 assay confirmed that the proliferation of CC cells was lower in cells transfected with pCMV3-MS4A1 and pCMV3-TNFRSF17 than in control cells (*P* < 0.05, **Figure 8A**). Transwell invasion assay revealed that the invasive capability of CC cells was substantially lower in the pCMV3-MS4A and pCMV3-TNFRSF17 groups than in the control group (*P* < 0.01, **Figure 8B**). Wound healing assay revealed that the migration ability of CC cells transfected with pCMV3-MS4A1 and pCMV3-TNFRSF17 was significantly lower than that of control cells 24 h and 48 h after wound formation (*P* < 0.01, **Figures 9A, B**).

## Discussion

CC causes more than 500,000 deaths annually and seriously threatens human health (29). Owing to the absence of evident symptoms in the early stage, patients with CC are often diagnosed at the middle and late stages of the disease (30). Therefore, early diagnosis of CC, accurate prediction of the prognosis of patients with CC and in-depth understanding of the underlying molecular mechanisms are of great clinical significance. CD8+ T cells serve as the major effector cells of anti-cancer immunity, and their high infiltration in TME can often inhibit the onset and progression of cancer. Therefore, high infiltration of CD8+ T cells can be used as an indicator to predict the prognosis of CC (31, 32), and elucidating its crucial role in the TME of CC is clinically significant for preventing the development of CC.

In this study, two genes, MS4A1 and TNFRSF17, which are associated with the high infiltration of CD8+ T cells, were screened *via* systematic bioinformatic methods, and their prognostic roles and biological functions in CC were investigated. Although MS4A1 is a primary biomarker of B cells, several studies have reported that it can also be expressed on T cells (33, 34). We found that MS4A1 was down-regulated in CC, of which low expression predicted the poor prognosis of CC, which indicates that MS4A1 may be a potential biomarker for predicting cancer progression. After further study, it was noticed that MS4A1 was related to lower cancer stem cell score, and our study revealed for the first time that MS4A1 may be a potential tumour suppressor gene in CC. Therefore, the specific role of MS4A1 was further explored in CC. When the expression of MS4A1 was interfered in CC cells, it was found that MS4A1 overexpression substantially inhibited the proliferation and invasion of CC cells. As far as we know, this study is the first *in vitro* experiment to show that MS4A1, as a tumour suppressor gene, can inhibit the progression of CC. However, the potential mechanism of CD8 + T cells participating in MS4A1-mediated tumour inhibitory effect remains to be further studied. In view of the fact that the correlation between TNFRSF17 and the high infiltration of CD8 + T cells in TME of CC was found through biological information analysis, the influence of TNFRSF17 on CC cells was further detected. When TNFRSF17 was overexpressed, the proliferation and migration of CC cells were decreased, which indicates that TNFRSF17 plays a role as a tumour suppressor gene in the process of CC disease. Previous literature had reported the prognostic role of TNFRSF17 in various cancers, including colon cancer, gastric cancer, lung cancer and ovarian cancer (35–38). In this study, CC patients with high expression of TNFRSF17 had better survival results, which was consistent with previous studies (35). These findings provide immune-related prognostic biomarkers for CC, and effective targets for clinical treatment of cancer.

Cancer immunotherapy has dramatically changed the paradigm of cancer treatment and has demonstrated its importance in the treatment of particular malignancies. Tumour immunotherapy includes cancer vaccines, cellular immunotherapy and ICIs (39). The emergence of immunotherapy has opened up new perspectives for the treatment of lung cancer. In patients with local non-small-cell lung cancer (NSCLC) (stage I - III), immunotherapy can help reduce the postoperative recurrence rate or improve the current clinical outcomes in the treatment of unresectable tumours (40). Tumour microenvironment includes tumour-killing cells such as CD8 + T cells, M1 macrophages and NK cells, carcinogenic immune cells and tumour-related macrophages. Immunosuppression induced by tumour microenvironment is still an important obstacle to limit the efficacy of immunotherapy in the treatment of NSCLC (41). It has been shown in the literature that ICI-CT combined therapy significantly improves the response and survival rate of NSCLC patients compared with chemotherapy (CT) alone. However, in squamous cell histology, PD-L1 expression was less than 50%. Among female NSCLC patients with liver metastasis and non-smoking history, there is low or no benefit. Therefore, the discovery of biomarkers may be beneficial to determine the most suitable candidate for the best ICI-CT combination (42). However, only a few patients with CC are sensitive to immunotherapy, with the majority of patients being unresponsive to it (43). Therefore, it is crucial to identify immune infiltration-related biomarkers to predict the responsiveness of patients with CC to immunotherapy. The TIDE algorithm allows accurate evaluation of the responsiveness of patients with cancer to immunotherapy. However, evaluation of TIDE scores requires an accurate assessment of the immune cell content in TME, which requires the assessment of the expression of many genes (28). In this study, TIDE scores were higher in patients with high MS4A1 and TNFRSF17 expression. In addition, the proportion of patients who were predicted to be more sensitive to immunotherapy was higher, which demonstrated the potential of MS4A1 and TNFRSF17 in predicting sensitivity to immunotherapy. Furthermore, high expression of immune checkpoints promotes the functionality of ICIs (43). In this study, patients with CC with high expression of MS4A1 and TNFRSF17 had high expression of immune checkpoints, which may have increased the sensitivity to immunotherapy. There are some limitations in this paper. Firstly, there may be a more complex regulatory mechanism between the gene expression of MS4A1 and TNFRSF17 reported in this study and CD8+T cell infiltration and immunotherapy. Secondly, due to the limitation of laboratory conditions, this study was only verified by *in vitro* experiments. Our follow-up team plans to focus on exploring the upstream regulation mechanism, downstream pathway of these two target genes and their in-depth relationship with immunity, and verify them through *in vivo* experiments.

## Conclusion

MS4A1 and TNFRSF17 are associated with high infiltration of CD8+ T cells in CC. Both genes are significantly downregulated in CC, and the prognosis of patients with CC with low expression of MS4A1 and TNFRSF17 is worse. Because MS4A1 and TNFRSF17 are substantially associated with immune cell infiltration in the TME of CC, they can serve as biomarkers for predicting the sensitivity of patients with CC to immunotherapy. In addition, these genes have therapeutic potential for the inhibition of CC progression.

## Data availability statement

The original contributions presented in the study are included in the article/**Supplementary Material**. Further inquiries can be directed to the corresponding author.

## Ethics statement

The study protocol was approved by the institutional review board of the First Affiliated Hospital of Harbin Medical University.

## Author contributions

YS and ZZ contributed equally to this paper, conceived and designed the study together. BZ contributed to the writing of the manuscript. WZ supervised the study. All authors contributed to the article and approved the submitted version.

## Conflict of interest

The authors declare that the research was conducted in the absence of any commercial or financial relationships that could be construed as a potential conflict of interest.

## Publisher’s note

All claims expressed in this article are solely those of the authors and do not necessarily represent those of their affiliated organizations, or those of the publisher, the editors and the reviewers. Any product that may be evaluated in this article, or claim that may be made by its manufacturer, is not guaranteed or endorsed by the publisher.

## Supplementary material

Supplementary Figure 1 Differential gene expression analysis **(A, B)** Heat maps demonstrating differentially expressed mRNAs in The Cancer Genome Atlas (TCGA) and Gene Expression Omnibus (GEO) datasets. The colour ranging from green to red indicates mRNA expression ranging from low to high, respectively. **(C, D)** Volcano plots demonstrating differentially expressed mRNAs in TCGA and GEO datasets. Green represents low mRNA expression in colon cancer (CC) samples, and red represents high mRNA expression in CC samples.

Supplementary Figure 2 Weighted gene co-expression analysis **(A)** Gene co-expression module of TCGA dataset. **(B)** Gene co-expression module of the Gene Expression Omnibus (GEO) dataset. **(C)** Correlation between TCGA gene modules and high and low CD8+ T-cell infiltration. **(D)** Correlation between the GEO gene modules and high and low CD8+ T-cell infiltration. **(E)** Intersection of differentially expressed genes from the brown modules of both datasets.

Supplementary Figure 3 PPI and the functional enrichment analysis of MS4A1 and TNFRSF17 **(A)** PPI network showed proteins interacting with MS4A1 and TNFRSF17 **(B, C)** GSEA analysis of MS4A1 and TNFRSF17
