# Supplementary figures and images for: CD8+ T cell-associated genes MS4A1 and TNFRSF17 are prognostic markers and inhibit the progression of colon cancer
Source: Front Oncol. 2022 Sep 20;12:941208. doi: 10.3389/fonc.2022.941208 (PMC9530608; doi:10.3389/fonc.2022.941208)

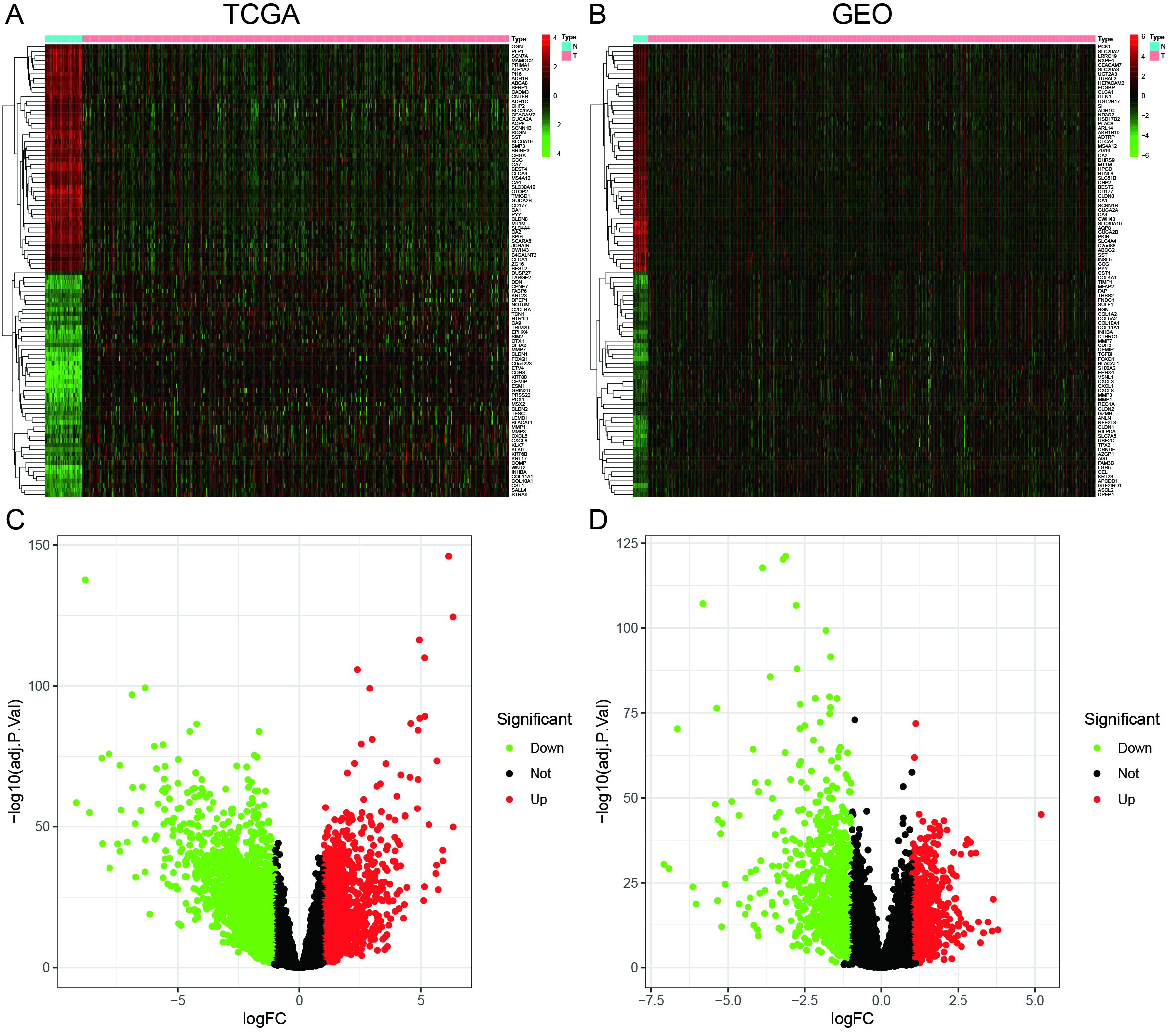

Supplement: Supplementary Figure 1 — Differential gene expression analysis (A, B) Heat maps demonstrating differentially expressed mRNAs in The Cancer Genome Atlas (TCGA) and Gene Expression Omnibus (GEO) datasets. The colour ranging from green to red indicates mRNA expression ranging from low to high, respectively. (C, D) Volcano plots demonstrating differentially expressed mRNAs in TCGA and GEO datasets. Green represents low mRNA expression in colon cancer (CC) samples, and red represents high mRNA expression in CC samples. [file Image_1.jpeg]

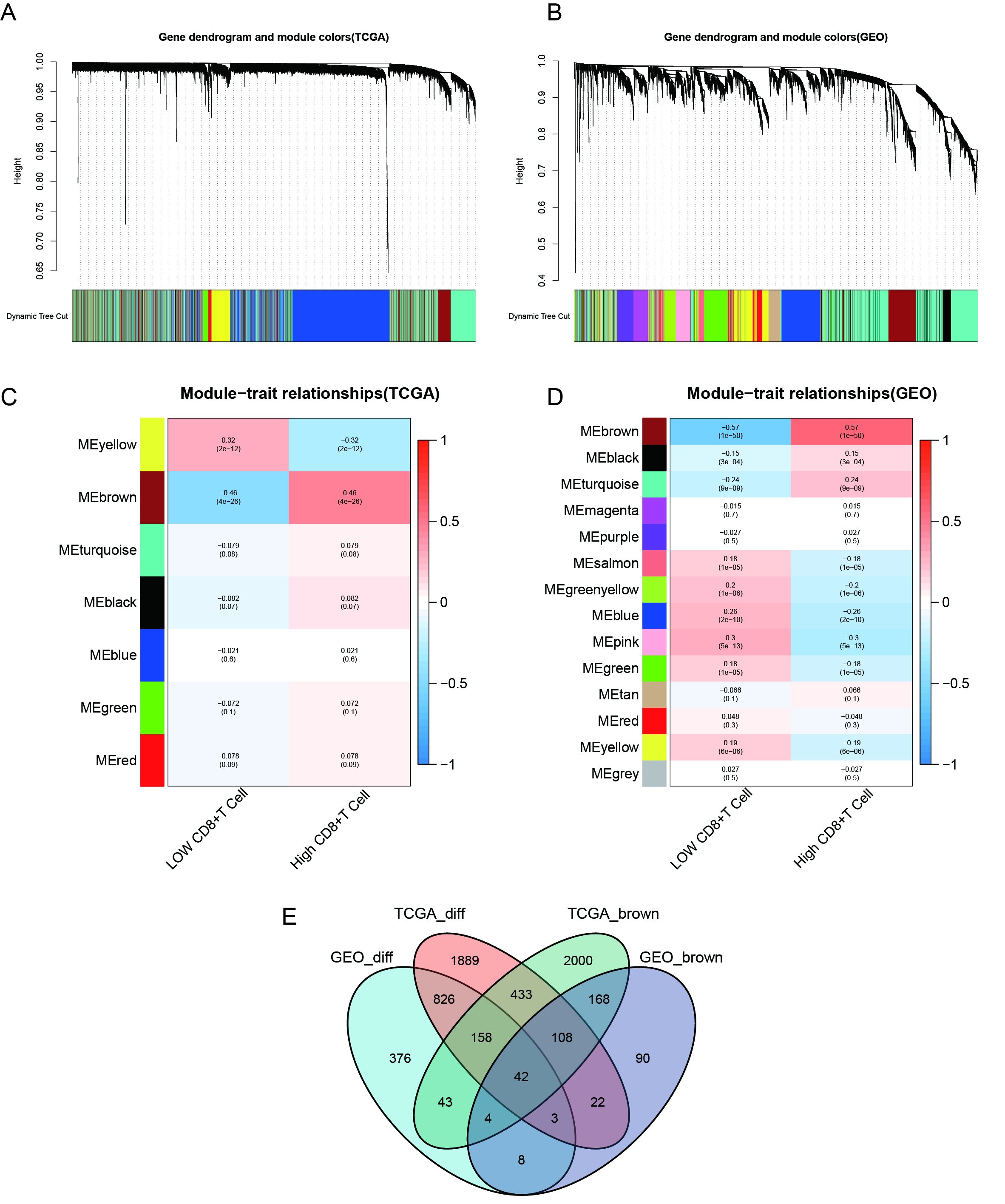

Supplement: Supplementary Figure 2 — Weighted gene co-expression analysis (A) Gene co-expression module of TCGA dataset. (B) Gene co-expression module of the Gene Expression Omnibus (GEO) dataset. (C) Correlation between TCGA gene modules and high and low CD8+ T-cell infiltration. (D) Correlation between the GEO gene modules and high and low CD8+ T-cell infiltration. (E) Intersection of differentially expressed genes from the brown modules of both datasets. [file Image_2.jpeg]

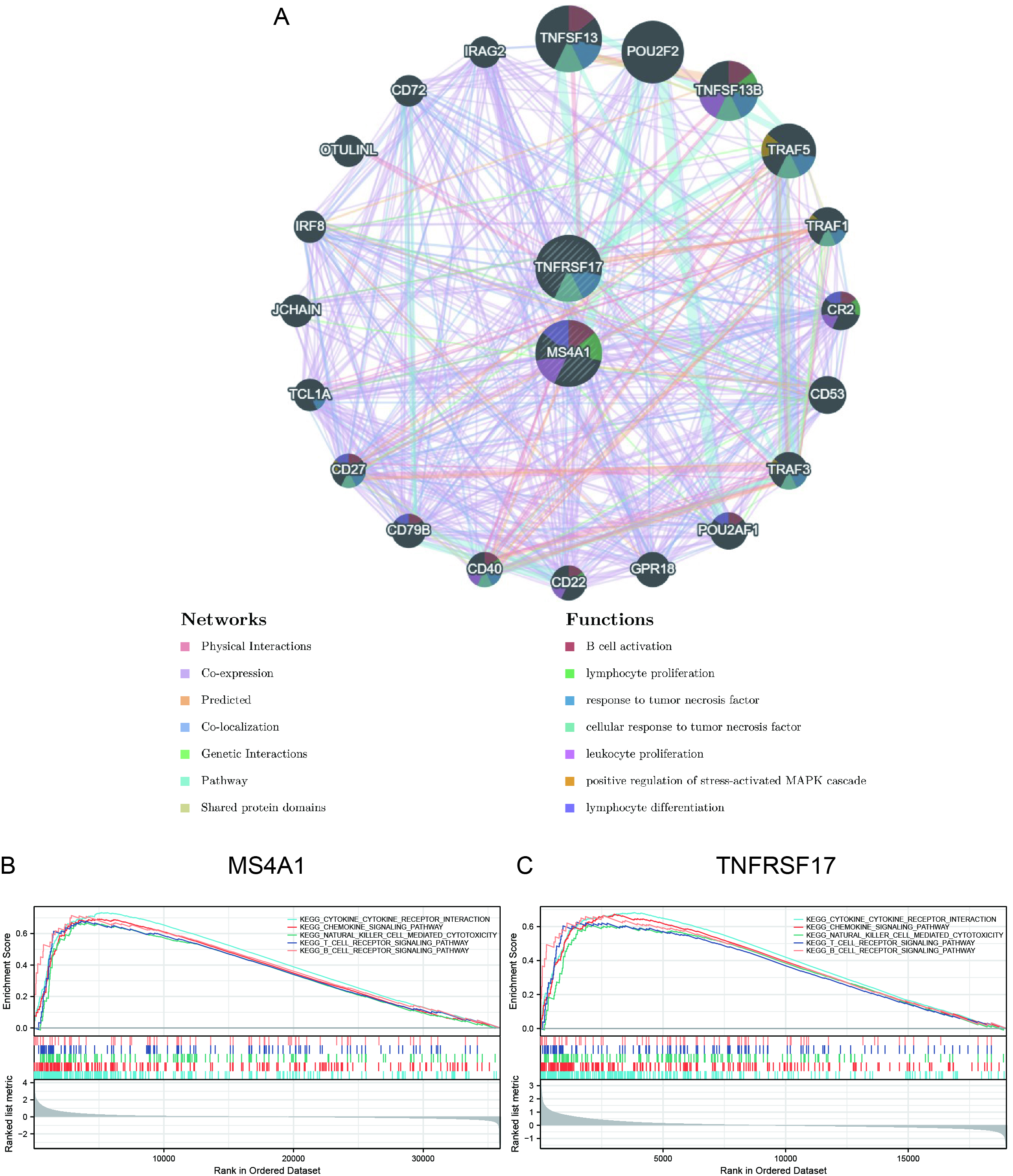

Supplement: Supplementary Figure 3 — PPI and the functional enrichment analysis of MS4A1 and TNFRSF17 (A) PPI network showed proteins interacting with MS4A1 and TNFRSF17 (B, C) GSEA analysis of MS4A1 and TNFRSF17 [file Image_3.jpeg]
